# National Prevalence and Trends of HIV Transmitted Drug Resistance in Mexico

**DOI:** 10.1371/journal.pone.0027812

**Published:** 2011-11-15

**Authors:** Santiago Avila-Ríos, Claudia García-Morales, Daniela Garrido-Rodríguez, Christopher E. Ormsby, Ramón Hernández-Juan, Jaime Andrade-Villanueva, Luz A. González-Hernández, Indiana Torres-Escobar, Samuel Navarro-Álvarez, Gustavo Reyes-Terán, For the Mexican HIV Molecular Epidemiology Project Group

**Affiliations:** 1 Centro de Investigación en Enfermedades Infecciosas, Instituto Nacional de Enfermedades Respiratorias, Mexico City, Mexico; 2 Unidad de VIH/SIDA, Hospital Civil de Guadalajara Fray Antonio Alcalde, Guadalajara, Jalisco, Mexico; 3 Universidad de Guadalajara, Guadalajara, Jalisco, Mexico; 4 Hospital General de Puebla, Puebla, Puebla, Mexico; 5 Facultad de Medicina, Benemérita Universidad Autónoma de Puebla, Puebla, Puebla, Mexico; 6 Hospital General de Tijuana, Tijuana, Baja California, Mexico; University of Nebraska – Lincoln, United States of America

## Abstract

**Background:**

Transmitted drug resistance (TDR) remains an important concern for the management of HIV infection, especially in countries that have recently scaled-up antiretroviral treatment (ART) access.

**Methodology/Principal Findings:**

We designed a study to assess HIV diversity and transmitted drug resistance (TDR) prevalence and trends in Mexico. 1655 ART-naïve patients from 12 Mexican states were enrolled from 2005 to 2010. TDR was assessed from plasma HIV *pol* sequences using Stanford scores and the WHO TDR surveillance mutation list. TDR prevalence fluctuations over back-projected dates of infection were tested. HIV subtype B was highly prevalent in Mexico (99.9%). TDR prevalence (Stanford score>15) in the country for the study period was 7.4% (95% CI, 6.2∶8.8) and 6.8% (95% CI, 5.7∶8.2) based on the WHO TDR surveillance mutation list. NRTI TDR was the highest (4.2%), followed by NNRTI (2.5%) and PI (1.7%) TDR. Increasing trends for NNRTI (p = 0.0456) and PI (p = 0.0061) major TDR mutations were observed at the national level. Clustering of viruses containing minor TDR mutations was observed with some apparent transmission pairs and geographical effects.

**Conclusions:**

TDR prevalence in Mexico remains at the intermediate level and is slightly lower than that observed in industrialized countries. Whether regional variations in TDR trends are associated with differences in antiretroviral drug usage/ART efficacy or with local features of viral evolution remains to be further addressed.

## Introduction

Antiretroviral therapy (ART) has radically decreased HIV-associated morbidity and mortality in countries where broad access to antiretroviral (ARV) drugs has been achieved. However, a wider availability of ART has led to increasing transmission of HIV variants with reduced susceptibility to ARV drugs [1], [2], [3], [4], [5], [6], [7], [8], [9]. Transmitted drug resistance (TDR) can reduce the efficacy of first-line ARV therapy, as complete suppression of HIV may be compromised [10]. The presence of resistance mutations in isolates from ARV-drug-naïve patients remains an important concern for the management of HIV infection, especially in the setting of resource-limited countries that have recently scaled-up ART access. Nevertheless, most patients in this setting are starting ART on potent regimens, possibly delaying transmission of drug-resistant HIV strains as compared with high-income countries, where ART scale-up began with suboptimal and lower-potency regimes [11]. This hypothesis is supported by the observation of stabilizing or decreasing tendencies in TDR in some developed countries during the last few years, which could be reflecting the more recent broad use of high-potency ART regimes [1], [12], [13], [14]. Ongoing TDR surveillance programs using comparable drug resistance definitions are necessary to guide worldwide efforts to improve treatment outcomes by supplying information to support education and prevention programs and promote the rational use of ARV drugs by clinicians and policy makers [11], [15], [16], [17].

Efforts to provide broad access to ART in Mexico started in 2001 with a universal access program, but it was until 2004 that coverage for persons without insurance was initiated [18]. Currently, all individuals who approach the Mexican Health System have access to ART either through the traditional social insurance program or the popular insurance system, introduced widely in the population by 2006 [19]. According to data from the National Centre for AIDS Prevention and Control (CENSIDA) and UNAIDS, by the end of 2009, 220,000 adults were estimated to live with HIV in Mexico from which 27% were receiving ART, 14% were under medical follow-up without ART and 59% may have been unaware of their HIV infection status or were not under medical follow-up in any public institution [18]. Considering this scenario, the extent to which TDR has spread in Mexico after nearly five years of broad access to ART remains an important issue to be assessed.

The remarkable genetic diversity of HIV has important implications for multiple aspects of the pandemic such as diagnostic and follow-up laboratory tests, candidate vaccine design, susceptibility to ART, transmission capability, virulence and disease progression [20]. Similarly to other parts of the North American epidemic, it is estimated that subtype B virus is highly predominant in Mexico [21], [22], [23], [24]. However, characterization of HIV molecular epidemiology in the country is incomplete, with the majority of existing studies being limited to small cohorts of infected individuals, or focusing on specific geographic areas and high-risk groups [24], [25], [26]. Recent studies have shown increasing trends in HIV diversity in the USA with nearly 4% of non-B viruses already circulating in the country [7], [27]. In Mexico, an increase in the circulation of non-B subtypes cannot be discarded either [24].

We established collaborations with several health centers and HIV clinics in 12 Mexican states, managed mainly by the Mexican Ministry of Health, to conduct the first large, prospective study to assess HIV TDR prevalence and trends at the national level. A cohort of 1655 ART-naïve patients was formed from 2005 to 2010. Plasma HIV RNA *pol* sequences were obtained and TDR was assessed. We observed intermediate TDR levels at the national level and important regional differences in TDR trends during the study period, suggesting several scenarios in the Mexican HIV epidemic and its management in the country.

## Materials and Methods

### Ethics Statement

Written informed consent was obtained for every participant before blood sample donation. The study was revised and approved by the Ethics Committee of the National Institute of Respiratory Diseases and was conducted according to the principles of the Declaration of Helsinki.

### Patients

**Figure 1:**
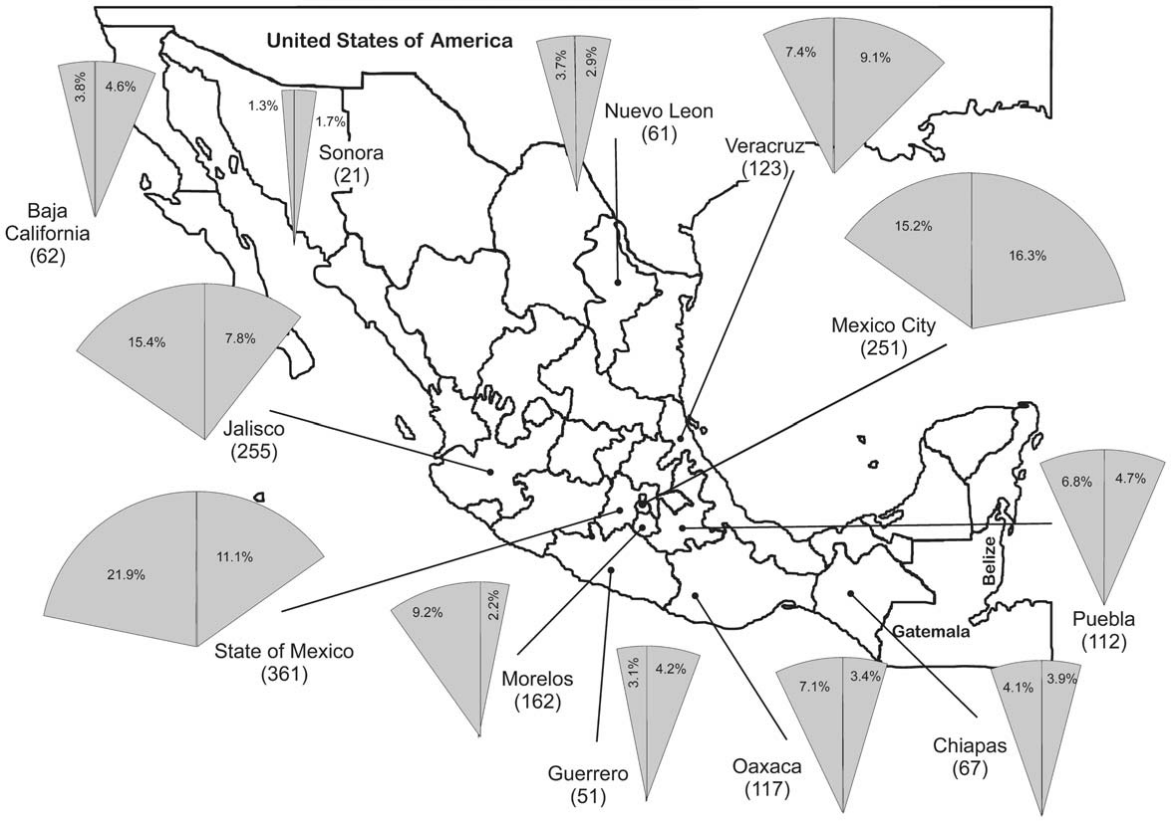
Geographic distribution of individuals participating in the study. 1655 ART-naïve individuals from 12 Mexican states were enrolled in the current study. The percentage of individuals from each state participating in the study is shown in the cake slice to the left. The proportion of national HIV infections reported for each participating state according to data from the National Centre for HIV/AIDS Prevention and Control (CENSIDA) [28] is shown in the cake slice to the right.

We established a national collaborative network between HIV clinics, state laboratories, regional hospitals and the National Institute of Respiratory Diseases (INER, a national third-level referral centre and one of the National Institutes of Health) to prospectively assess HIV molecular epidemiology and TDR prevalence and trends in Mexico. Twelve Mexican states that together account for more than 80% of all the officially registered HIV infections in Mexico [28] participated in this study (Figure 1). Newly diagnosed, ART-naïve HIV patients and individuals previously diagnosed that had not started ART were enrolled from 2005 to 2010. After giving written informed consent, patients donated a single peripheral blood sample. Demographic data was collected through direct application of a questionnaire before sample donation. All blood samples were sent to and processed at the INER in Mexico City within the following 24 hours from collection. Plasma viral load assays, CD4+ T cell counts and HIV genotyping and TDR analyses were performed for each participating individual. Results were sent back to the corresponding health centres for patient clinical follow-up.

### HIV sequencing and genotypic drug resistance testing

HIV RNA *pol* sequences were obtained using ViroSeq HIV-1 Genotyping System (Celera Diagnostics, Alameda, CA), according to the manufacturer's specifications, from a fragment of the viral *pol* gene including the whole protease (PR) and 334 codons of the reverse transcriptase (RT). Bulk sequences were obtained with a 3100-Avant Genetic Analyzer (Applied Biosystems, Foster City, CA). Sequencing PCRs were carried out with 7 different primers to assure that the whole genomic region was covered with at least two sequences. Sequences were assembled, aligned to the HXB2 consensus, and manually edited using the ViroSeq v2.7 software provided by the manufacturer.

Genotypic drug resistance analyses were carried out with the Stanford HIV Drug Resistance Database algorithm, using the HIVdb program [29], [30]. The presence of resistance was defined according to Stanford score (SS) ranges as follows: 0–9: susceptible; 10–14: potential low-level resistance; 15–29: low-level resistance; 30–59: intermediate resistance; 60 or higher: high-level resistance. All samples were analyzed at the same time using the last algorithm update available at the time of writing (Version 6.0.11). Additionally, genotypic drug resistance was defined according to the presence of drug resistance mutations of the list for HIV TDR surveillance proposed and periodically updated by the WHO [31].

### TDR surveillance using WHO TDR thresholds

We retrospectively applied the WHO TDR threshold method for global HIV TDR surveillance to the cohort [11], [32]. Patients under 25 years of age and CD4+ T cell counts over 500 cells/µl were selected in order to determine TDR thresholds mainly with individuals infected within a 3-year period before the time of enrolment. HIV genotypes from the first 47 patients to be subsequently enrolled that fulfilled the eligibility criteria mentioned above, were considered for this sub-analysis as previously described [11]. The presence of TDR was defined with the WHO HIV TDR surveillance list [31]. HIV TDR thresholds were established for twelve-month periods and TDR prevalence was categorized as low (<5%), moderate (≥5%–≤15%), or high (>15%).

### Statistical Analyses

TDR prevalence fluctuations were examined over the estimated time of HIV infection by graphical methods using moving average with a six-month window. In order to use a date variable closer to the actual time of HIV infection, a model suggested by Mellors et al. [33] was used for estimating the delay between HIV infection and time of diagnosis in the Mexican cohort. Significance was ascertained with Poisson regression. An ordinal logistic regression model (lrm from package Design ver 2.3–0) was used to test the variation of Stanford Scores as a function of CD4+ T cell count and viral load. All analyses were carried out with R statistical software version 2.12.0.

### HIV subtyping and phylogenetic analyses

HIV subtyping was performed with REGA Subtyping Tool v2.0 [34], [35], available on line. Recombination was confirmed using RIP HIV Recombination Identification Program [36], available on line. We used PhyML ver 2.4.4 to estimate the maximum likelihood phylogeny of protease and RT sequences separately, with 100 replicates for bootstrap analysis, and a GTR+I+gamma base optimization model. The resulting trees were explored with the R package “ape”, version 2.7–3 [37], to look for possible transmission clusters and geographical effects.

We selected the most frequent TDR mutations for PI, NRTI and NNRTI (A71V, G333E and K103R, respectively) to examine transmission patterns of specific mutations in the trees. In order to control for the possibility that the branch convergence observed was due to having the TDR mutation itself, we selected tree tips (patients) that had the TDR mutation studied and bootstrapping support in the tree of the other gene; that is, we looked for bootstrap values for patients with K103R and G333E in the protease tree, and for A71V in the RT tree, since these changes were not used to build each of those trees.

## Results

### TDR levels and HIV diversity in Mexico

We prospectively analyzed for TDR the HIV protease-RT sequences of 1655 ART-naïve individuals from 12 Mexican states, between 2005 and 2010. The samples collected came from Mexican states that together account for 82.8% of all the officially reported cases in the country [28]. In general, the proportion of patients enrolled for each state in the study was similar to the proportion of the total number of infections officially reported for that state, although some states were over or under represented in the cohort (Figure 1).

The Mexican cohort included a high proportion of individuals in late stages of HIV disease, with a median CD4+ T cell count of 228 cells/µl (Table 1). This characteristic late detection of HIV infection has been previously recognized in Mexico [18]. Nearly half of the participating individuals were diagnosed with CD4+ T cell counts under 200 cells/µl, and 18% under 50 cells/µl. Four out of five patients enrolled were male and the mean age at enrolment was 32.5 years (Table 1). No increasing or decreasing trends were seen in the proportion of females enrolled during the study period (p = 0.11).

**Table 1:** Demographic and clinical characteristics of individuals with and without TDR in the Mexican cohort.

	Total	Susceptible^a^	TDR (SS≥10)	TDR (SS≥15)	TDR (SS≥30)	TDR (SS≥60)
n	1655	1426	228	122	78	41
Mean Age (years, [IQR])	32.5 [25∶38]	32.6 [25∶38]	32.1 [25∶38]	31.9 [25∶38]	30.4 [24.5∶35]	29.5 [23∶35]
Proportion of Females [n (%)]	337 (20.4)	294 (20.6)	43 (18.8)	18 (14.8)	8 (10.3)	5 (12.2)
Median Viral Load, (RNA copies/mL, [IQR])	74,474 [21,295∶251,387]	74,270 [21,607∶244,000]	79,510 [20,105∶303,703]	57,189 [18,495∶293,059]	39,729 [10,828∶234,000]	51,538 [17,417∶369,507]
Median CD4+ T cell count, (cells/µL, [IQR])	227.8 [82.4∶417.6]	227.9 [83∶422]	227.6 [72.3∶390]	249 [71.5∶439]	253 [83.2∶482.4]	253 [103∶456.8]

a Individuals with Stanford Scores lower than 10. TDR – Transmitted Drug Resistance; SS – Stanford Score; IQR – Interquartile Range.

An unexpectedly high frequency of clade B viruses was found in the Mexican cohort, with 99.9% (1653/1655) of the viruses belonging to this subtype. Only two non-B sequences were found, belonging to the CRF12-BF and the CRF06-cpx circulating recombinant forms. The patients presenting these recombinant viruses referred having high-risk activities for HIV infection in South America and Europe respectively.

A global TDR prevalence of 7.4% (122/1655, 95% confidence interval [CI] 6.2∶8.8) to any ARV drug was found for the whole study period using Stanford scores (SS) with a threshold value of 15 (at least low-level ARV drug resistance). This definition of TDR was comparable to the one based on the WHO TDR surveillance mutation list [31], which predicted a general TDR prevalence for any ARV drug of 6.8% (113/1655, 95% CI 5.7∶8.2) (Table 2). Using a Stanford score threshold of 15, the prevalence of TDR resistance to nucleoside RT inhibitors (NRTIs) was the highest (69/1655, 4.2%, 95% CI 3.3∶5.3), followed by non-nucleoside RT inhibitors (NNRTIs) (42/1655, 2.5%, 95% CI 1.9∶3.4) and protease inhibitors (PIs) (28/1655, 1.7%, 95% CI 1.1∶2.5) (Table 2). TDR to NNRTIs was lower using the WHO TDR surveillance mutation list definition compared to the Stanford algorithm, which takes into account the additive effect of minor mutations such as K101Q, K103R and V179D for global resistance levels. High-level ARV drug resistance (SS≥60) was more frequently observed for NNRTIs (26/1655, 1.6%) than for PIs (14/1655, 0.8%) or NRTIs (6/1655, 0.4%) (Table 2). The prevalence of TDR to multiple drug classes was low with 0.8% (13/1655) and 0.1% (2/1655) of patients showing resistance to two or three drug classes respectively.

**Table 2:** TDR prevalence in a cohort of 1655 ART-naïve Mexican individuals from 12 states.

Drug Class	TDR Level [n (%, 95% confidence interval)]^a^				
	SS≥10	SS≥15	SS≥30	SS≥60	WHO
Any ARV Drug	228 (13.8, 12.2∶15.6)	122 (7.4, 6.2∶8.8)	78 (4.7, 3.8∶5.9)	41 (2.5, 1.8∶3.4)	113 (6.8, 5.7∶8.2)
NRTI	80 (4.8, 3.9∶6.0)	69 (4.2, 3.3∶5.3)	26 (1.6, 1.0∶2.3)	6 (0.4, 0.1∶0.8)	69 (4.2, 3.3∶5.3)
PI	51 (3.1, 2.3∶4.1)	28 (1.7, 1.1∶2.5)	28 (1.7, 1.1∶2.5)	14 (0.8, 0.5∶1.5)	29 (1.8, 1.2∶2.5)
NNRTI	118 (7.1, 6.0∶8.5)	42 (2.5, 1.9∶3.4)	32 (1.9, 1.3∶2.8)	26 (1.6, 1.0∶2.3)	31 (1.9, 1.3∶2.7)

a Data shown is number, percentage and 95% confidence interval. SS – Stanford Score; ARV – Antiretroviral; NRTI – Nucleoside Reverse Transcriptase Inhibitors; PI – Protease Inhibitors; NNRTI – Non-Nucleoside Reverse Transcriptase Inhibitors.

It is important to note that a lower SS cut-off provides greater sensitivity for identifying individuals with true phenotypic resistance, but lower specificity, capturing more false positives. However, as stated above, a SS cut-off of 15 best correlates with the use of the WHO TDR mutation list for defining resistance and even if phenotypic resistance were not present, the existence of TDR mutations with low SS could be indicative of selection by ARV drugs and could be relevant to study TDR transmission.

We retrospectively applied the WHO TDR threshold method for TDR surveillance in the Mexican cohort. Selecting individuals under 25 years of age and ≥500 CD4+ T cells/µl, TDR levels in Mexico were in the moderate range (≥5%–≤15%) for 2008 and 2009. This categorization persisted when considering individuals under 25 years of age and >350 CD4+ T cells/µl in six-month period analyses for both years.

**Figure 2:**
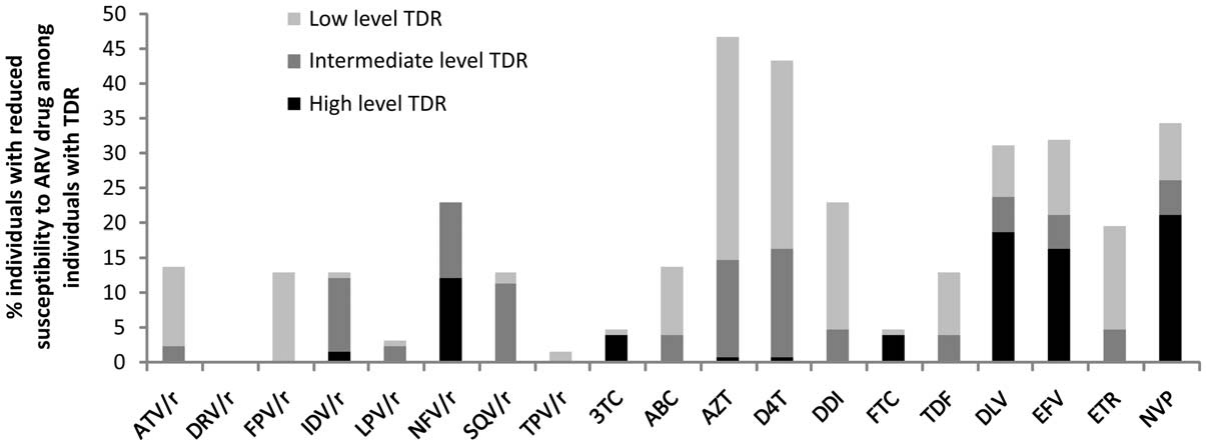
Antiretroviral drug (ARV) resistance levels to the most common antiretroviral drugs among individuals with transmitted drug resistance (TDR). The levels of ARV drug resistance in the 122 of 1655 individuals with TDR in the Mexican cohort are shown. Low-level resistance corresponds to a Stanford Score (SS) between 15 and 29, intermediate-level resistance to a SS between 30 and 59, and high-level resistance to a SS equal to or over 60.

Among individuals with TDR, high-level resistance (SS≥60) was observed more frequently to nevirapine (26/122, 21.3%), delavirdine (23/122, 18.9%), efavirenz (20/122, 16.4%), nelfinavir (15/122, 12.3%), emtricitabine (5/122, 4.1%) and lamivudine (5/122, 4.1%), while zidovudine (57/122, 46.7%) and stavudine (53/122, 43.4%) were the ARV drugs most frequently affected by at least low-level TDR (Figure 2).

Most NRTI TDR cases were associated with the presence of thymidine analogue mutations (TAMs), with 70.4% (57/81) of the viruses with any degree of NRTI resistance (SS≥10) expressing at least one TAM. From all the NRTI TDR cases 1.2% (1/81), 11.1% (9/81), and 8.6% (7/81) showed the combination of 4, 3, and 2 TAMs respectively, and 49.4% (40/81) presented a single TAM. Among individuals with more than 1 TAM, 70.6% (12/17) showed a type 1 mutation pathway (M41L, L210W, T215 revertants), while the rest showed a type 2 mutation pathway (D67NG, K70R, T215 revertants, K219RQEN). Interestingly, 44.4% (36/81) of the NRTI resistance cases showed the presence of RT T215 revertants, suggesting ARV drug-dependent selection and transmission of drug resistance mutations. The M184VI mutation was detected in 5 individuals (0.3% of the whole cohort, 7.2% of individuals with NRTI TDR) while mutations of the Q151M complex were not observed (Table 3).

**Table 3:** Prevalence of transmitted ARV drug resistance mutations in a cohort of 1655 Mexican individuals from 12 states.

Protease inhibitors (PI)					Nucleoside RT inhibitors (NRTI)					Non-nucleoside RT inhibitors (NNRTI)				
Mutation	Frequency in cohort [n (%)]		Frequency in individuals with TDR [n (%)]*		Mutation	Frequency in cohort [n (%)]		Frequency in individuals with TDR [n (%)]*		Mutation	Frequency in cohort [n (%)]		Frequency in individuals with TDR [n (%)]*	
L10IV	204	(12.3)	9	(32.1)	M41L	19	(1.1)	19	(27.5)	A98G	3	(0.2)	1	(2.4)
L10F	2	(0.1)	0	(0.0)	M41R	1	(0.1)	0	(0.0)	L100I	0	(0.0)	0	(0.0)
V11I	4	(0.2)	0	(0.0)	E44D	4	(0.2)	1	(1.4)	K101Q	21	(1.3)	5	(11.9)
L23I	0	(0.0)	0	(0.0)	A62V	1	(0.1)	0	(0.0)	K101N	1	(0.1)	1	(2.4)
L24I	0	(0.0)	0	(0.0)	K65R	0	(0.0)	0	(0.0)	K101E	4	(0.2)	4	(9.5)
D30N	1	(0.1)	1	(3.6)	D67T	1	(0.1)	0	(0.0)	K103NS	18	(1.1)	18	(42.9)
V32I	0	(0.0)	0	(0.0)	D67H	1	(0.1)	0	(0.0)	K103R	119	(7.2)	5	(11.9)
L33F	7	(0.4)	1	(3.6)	D67NG	8	(0.5)	8	(11.6)	V106A	0	(0.0)	0	(0.0)
E35G	2	(0.1)	0	(0.0)	D67E	0	(0.0)	0	(0.0)	V106M	0	(0.0)	0	(0.0)
K43T	8	(0.5)	1	(3.6)	T69A	11	(0.7)	0	(0.0)	V108I	15	(0.9)	4	(9.5)
M46IL	12	(0.7)	12	(42.9)	T69D	5	(0.3)	5	(7.2)	E138KQ	2	(0.1)	1	(2.4)
I47A	0	(0.0)	0	(0.0)	T69ins	0	(0.0)	0	(0.0)	E138GA	27	(1.6)	3	(7.1)
I47V	0	(0.0)	0	(0.0)	T69N	12	(0.7)	1	(1.4)	V179AT*	1	(0.1)	0	(0.0)
G48VM	0	(0.0)	0	(0.0)	T69C	0	(0.0)	0	(0.0)	V179D	44	(2.7)	7	(16.7)
I50L	0	(0.0)	0	(0.0)	T69I	1	(0.1)	0	(0.0)	V179E	21	(1.3)	1	(2.4)
I50V	0	(0.0)	0	(0.0)	T69G	0	(0.0)	0	(0.0)	V179F	1	(0.1)	1	(2.4)
F53L	0	(0.0)	0	(0.0)	T69S	31	(1.9)	4	(5.8)	Y181IV	0	(0.0)	0	(0.0)
F53Y	0	(0.0)	0	(0.0)	K70G	0	(0.0)	0	(0.0)	Y181C	4	(0.2)	4	(9.5)
I54VA	0	(0.0)	0	(0.0)	K70N	0	(0.0)	0	(0.0)	Y188L	2	(0.1)	2	(4.8)
I54L	0	(0.0)	0	(0.0)	K70R	1	(0.1)	1	(1.4)	Y188H	0	(0.0)	0	(0.0)
I54M	0	(0.0)	0	(0.0)	K70E	0	(0.0)	0	(0.0)	Y188C	0	(0.0)	0	(0.0)
I54ST	0	(0.0)	0	(0.0)	L74I	1	(0.1)	1	(1.4)	G190S	1	(0.1)	1	(2.4)
Q58E	17	(1.0)	1	(3.6)	L74V	0	(0.0)	0	(0.0)	G190A	3	(0.2)	3	(7.1)
A71IVT	406	(24.5)	14	(50.0)	V75L	10	(0.6)	1	(1.4)	G190E	0	(0.0)	0	(0.0)
G73CSTA	0	(0.0)	0	(0.0)	V75A	0	(0.0)	0	(0.0)	G190C	0	(0.0)	0	(0.0)
T74S	2	(0.1)	1	(3.6)	V75T	0	(0.0)	0	(0.0)	P225H	2	(0.1)	2	(4.8)
L76V	0	(0.0)	0	(0.0)	V75S	0	(0.0)	0	(0.0)	F227L	1	(0.1)	1	(2.4)
V82A	3	(0.2)	3	(10.7)	V75M	2	(0.1)	2	(2.9)	M230L	1	(0.1)	1	(2.4)
V82F	0	(0.0)	0	(0.0)	F77L	0	(0.0)	0	(0.0)	K238T	2	(0.1)	2	(4.8)
V82T	0	(0.0)	0	(0.0)	Y115F	0	(0.0)	0	(0.0)	Y318F	1	(0.1)	1	(2.4)
V82S	0	(0.0)	0	(0.0)	F116Y	0	(0.0)	0	(0.0)					
V82M	0	(0.0)	0	(0.0)	V118I	78	(4.7)	6	(8.7)					
V82C	0	(0.0)	0	(0.0)	Q151M	0	(0.0)	0	(0.0)					
V82L	0	(0.0)	0	(0.0)	M184VI	5	(0.3)	5	(7.2)					
N83D	0	(0.0)	0	(0.0)	L210W	8	(0.5)	8	(11.6)					
I84VAC	0	(0.0)	0	(0.0)	T215Y	2	(0.1)	2	(2.9)					
I85V	2	(0.1)	1	(3.6)	T215F	0	(0.0)	0	(0.0)					
N88D	2	(0.1)	2	(7.1)	T215CDESIV	36	(2.2)	36	(52.2)					
N88S	0	(0.0)	0	(0.0)	K219QEN	6	(0.4)	6	(8.7)					
L90M	14	(0.8)	14	(50.0)	K219R	5	(0.3)	5	(7.2)					
					G333D	2	(0.1)	0	(0.0)					
					G333E	128	(7.7)	4	(5.8)					

*Frequency in individuals with resistance to any drug of the corresponding drug class (Stanford Score >15).

The NNRTI resistance mutation K103NS was present in 1.1% (18/1655) of the cohort and explained about 70% of high-level NNRTI resistance cases (Table 3). Low-level resistance cases were generally explained by the combination of minor mutations such as K101QE, K103R and V179D. Remarkably, the K103R minor mutation was found in 7.2% (119/1655) of all the individuals included in the study and in 11.9% (5/42) of individuals with NNRTI TDR (Table 2). This is a significantly higher occurrence than the 2% expected in ART naïve, clade B-infected cohorts and the 3.5% expected in NNRTI-experienced cohorts [38].

The presence of L90M explained all the cases of high-level PI TDR (14/14), while M46IL explained most of the intermediate-level PI TDR cases (12/14). Characteristically, 64.3% (18/28) of PI TDR cases showed the presence of more than one PI resistance-associated mutation (range 2 to 6). Interestingly, the polymorphic A71T/V mutation in protease was found in nearly one fourth of the individuals enrolled in the study (406/1655), compared with the 2–3% expected in PI-untreated populations [38] and in 50% (14/28) of individuals with PI TDR. Similarly, the V10IV mutation was observed in 12.3% (204/1655) of all the individuals in the cohort (with an expected prevalence of 5–10% in untreated persons [38]) and 32.1% (9/28) of individuals with PI TDR (Table 3).

Univariate analyses showed no differences in demographic characteristics between subjects with and without TDR (Table 1). Nevertheless, a logistic regression model showed that individuals with any TDR mutation had a tendency to present higher viral loads (p = 0.0374) (Table S1). Although stratification in SS apparently reflects lower viral loads associated with higher scores (Table 1), the number of subjects in each strata also decreases, making the patients with SS between 10 and 15 represent almost half of all the TDR cases, and therefore contributing more statistical weight to the model. Interquartile ranges in Table 1 also convey that the upper boundary of viral load is mostly higher in individuals with TDR than in individuals with susceptible viruses.

No differences were found in general TDR levels between the Mexican states included in the study. However, different clinical and demographic characteristics such as median plasma viral load, median CD4+ T cell count, mean age and percentage of females were observed in the subjects enrolled in different geographical regions, suggesting different epidemiological scenarios and clinical management of the infection in different areas of the country (Table S2).

### TDR trends in Mexico

Since 83.2% of the individuals in our cohort were diagnosed within 3 months prior to blood sample collection, and given the generally advanced stage of HIV disease at diagnosis (Table 1), there was a large gap between infection time and time of diagnosis. In order to use a date variable closer to actual HIV infection time, we used the model suggested by Mellors et al. [33] for estimating the delay between infection and diagnosis. Since the model assumes a CD4+ T cell count of 800 cells/uL at the time of infection, we used time of diagnosis for all values > = 800 to avoid estimation of infection dates later than HIV+ diagnosis dates. The back-projected dates derived from this model showed that patients in the cohort were infected between April 1991 and February 2010. Half of the patients were probably infected before August 2001 (interquartile range: October 1998 to February 2003).

**Figure 3:**
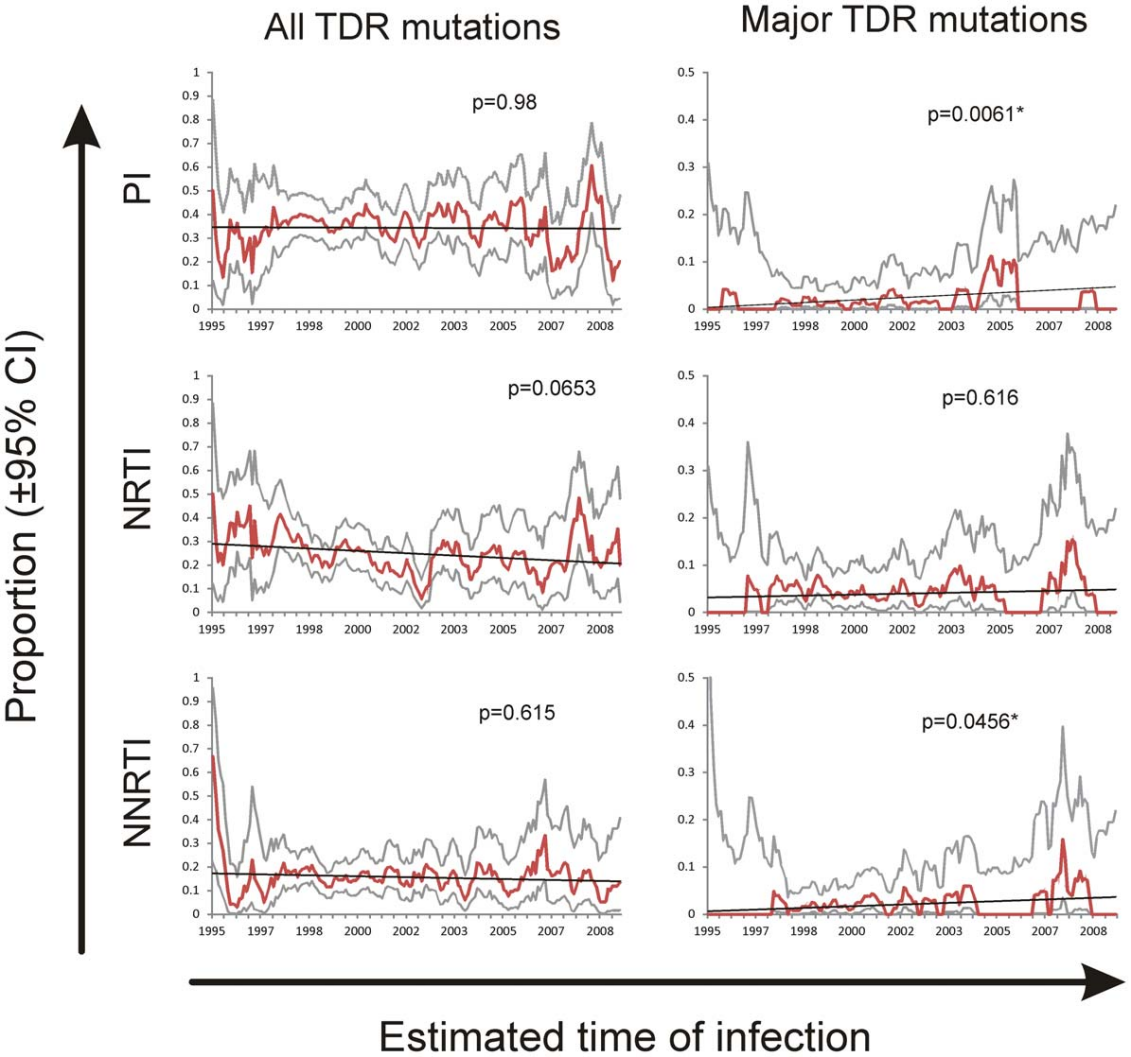
Transmitted drug resistance (TDR) trends in Mexico at the national level. Approximate dates of infection were estimated using a model described by Mellors et al [33]. TDR fluctuations were examined over the back-projected dates of infection by graphical methods using moving average with a six-month window. Significance was assessed with Poisson regression. Trends considering all TDR mutations and only major TDR mutations are shown.

Using a graphic moving-average method over the estimated HIV infection dates, a significant increasing trend was observed at the national level, when considering NNRTI major TDR mutations alone (p = 0.0456), and PI major TDR mutations alone (p = 0.0061) (Figure 3). No significant TDR trends were observed in regional analyses for NNRTIs or for PIs (Figure S1). Although a significant decreasing trend at the national level was not apparent for NRTIs (p = 0.0653), a strong decreasing trend in NRTI major TDR mutation frequency was observed in the Northwest region of the country, including a large proportion of individuals from the border city of Tijuana (p = 0.0074) (Figure S1).

Additionally, 14 TDR mutations showed trends of higher or lower prevalence in specific regions (p<0.05), from which 2 remained significant after correction for multiple comparisons (q<0.2) (Table S3); i.e. K103R with a higher prevalence in the Northeast and G333E with a higher prevalence in the West.

### Phylogenetic analyses

**Figure 4:**
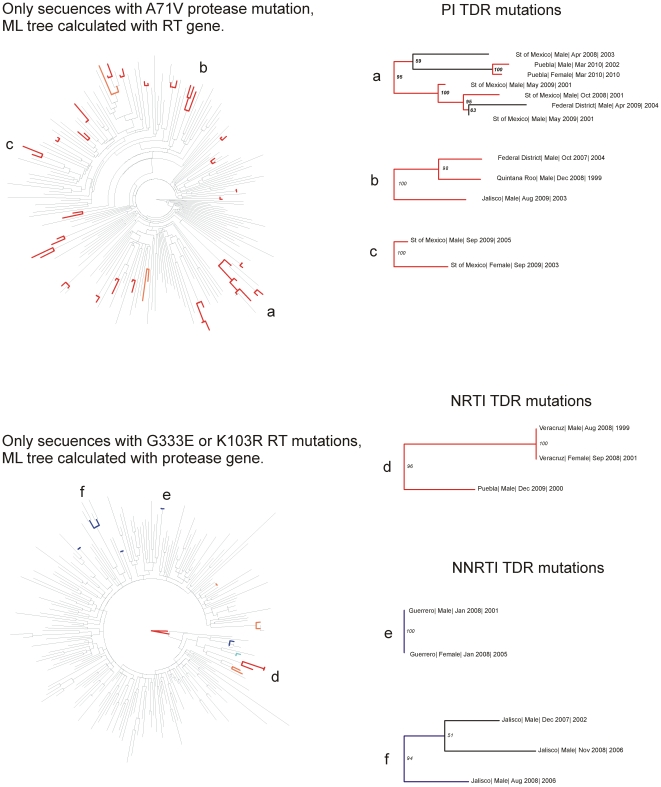
Sub-trees for specific protease and RT TDR mutations. Sub-trees were formed by selecting the patients that had a specific TDR mutation in one of the two genes, from the complete tree based on the other gene (see text for details). Zoom-ins on representative branches are shown on the right, expanding the information on the patients: State | Gender | date of sample collection | probable date of infection. Several branches (e.g. c and e) showed probable transmission pairs, while others formed small clusters with pairs within them (a and d). b and d show cases of widely circulating viruses with TDR mutations. Red lines show branches with boostrap values >90% for A71V and G333E, and blue lines for K103R. Lighter shades show bootstrap support >80%.

In order to establish the existence of possible TDR transmission clusters, we estimated maximum likelihood phylogenies for protease and RT sequences from all the participating individuals (Figure S2). The resulting trees were explored with R package “ape”. We observed scarce support for inner nodes, but several strongly related sequences in pairs or small clusters towards the tips of the branches. This suggests a high similarity between the sequences in the cohort, with no large geographical effects. Sequences with some minor TDR mutations appeared to form large clusters (Figure S2). Within these clusters, probable transmission pairs were detected, with sequences from the same geographical regions and close genetic distances. However, we also found well supported nodes that included sequences from distant geographical regions and large genetic distances, possibly suggesting a wider circulation of these mutations at a population level (Figure 4).

## Discussion

We present results from the largest national study to date assessing HIV molecular epidemiology and TDR prevalence and trends in Mexico. The study included states that together report more than 80% of all the infection cases in Mexico (Figure 1). Taking the 7.4% prevalence of TDR in the whole sample as an effect size, 80% power, and 0.05 significance, we estimated that a sample size of 1434 subjects was necessary for testing proportions. Considering that the study cohort was composed of 1655 individuals, a high representativity was achieved. Indeed, the Mexican Cohort reflected the previously characterized late detection of HIV infection, the lower prevalence of infection cases in females (18% of the officially reported cases) and the higher prevalence of the infection in the 30–44 age group (48% of the officially reported cases) (Table 1) [28].

Only 0.1% of the circulating viruses were identified as non-B subtypes. This prevalence is remarkably lower that that observed in the USA and Canada [7], [8], even after considering the geographic proximity and migratory efflux between these countries.

TDR prevalence in the country was shown to be at an intermediate range, according to WHO thresholds, and was slightly lower than that observed in some industrialized countries [1], [4], [6], [7], [14]. Although broad access to ART has been functional in Mexico for five years, the fact that nearly 60% of infected persons may be unaware of their serological status [18] could explain a slower spread of TDR in the Mexican setting, as less than half of the individuals who need ART would actually be receiving it [39]. A recent study by Wheeler and others [7] reported higher TDR levels in the USA compared to those observed in Mexico in the present study for similar time periods. Different TDR mutation patterns were also apparent in both countries: For PIs, M46IL (43% vs 21%; p = 0.0112, q = 0.1915) and L90M (50% vs 27%; p = 0.0056, q = 0.1911) were more prevalent in Mexico; for NRTIs, T215 revertants were more prevalent in Mexico (52% vs 36%, p = 0.0011, q = 0.0171) and T69N was more prevalent in the USA (1% vs 22%, p = 0.0012, q = 0.0171). Although differences exist in the design of both studies, these observations may reflect real epidemiological differences in TDR trends in the USA and Mexico. Comparisons in HIV TDR between the two countries are interesting given their geographical proximity, characteristic migratory patterns and differences in HIV disease management and policies. Taken together, these observations suggest different scenarios for HIV TDR in the two countries and have implications for HIV/AIDS management in the region as they might reflect a relatively closed contact network among migrants that acquire the infection abroad due to increased risk behaviour [40].

It is noteworthy that the CD4+ T cell counts of the newly diagnosed individuals were fairly low, with approximately half below 200 cells/uL (Table 1). Thus, considering that individuals in the Mexican setting frequently wait until they have symptoms to receive a positive HIV diagnosis, then individuals in our cohort could potentially reflect TDR several years earlier when they were likely to be infected. If TDR is related to calendar time reflecting scaling up of broad access to ART that started in 2001, then the TDR prevalence represented by this population might not be reflecting the current TDR prevalence as the current newly infected individuals are under-represented in the study population. In order to assess this issue, we used a previously reported model to estimate the delay between HIV infection and diagnosis [33]. HIV infection dates were back projected and TDR trends in time estimated. National TDR trends were stable when considering all TDR mutations within the period of 1994 to 2010, comprising the back-projected dates of infection of the individuals in the cohort. However, significant increasing trends were apparent for NNRTI and PI major mutations, as expected and observed in other countries that have implemented broad-access programs to ARV therapy [2], [6], [7], [11]. Nevertheless, a stable national trend and a decreasing trend in the Northwest for NRTI TDR are consistent with observations in other countries that report reductions in TDR prevalence, which may be associated with the broad use of high-potency, first-line ART regimes [1], [12], [14]. Indeed, only four high-potency ARV drug combinations account for half of all the prescribed schemes in Mexico, namely TDF + FTC + EFV (20.6%), ZDV + 3TC + EFV (14.8%), TDF + FTC + LPV/r (7.3%), and TDF + FTC + ATZ/r (5.3%) [39]. It is noteworthy that the majority of ARV drug combinations prescribed as first-line ART regimens in Mexico do not contain PIs. Thus, it is possible that the increasing trend in PI TDR observed in the present study may be partly associated with increasing use of this ARV drug family in second-line/salvage ART regimens. Although regional differences in TDR trends were scarce in the present study, the existence of multiple scenarios in HIV management and molecular epidemiology within the country cannot be discarded, and will need further assessment.

Several questions remain about the origin of TDR in the Mexican setting. According to data from the National System for ARV Drug Management, Logistics and Surveillance (SALVAR) at the National Centre for HIV/AIDS Prevention and Control (CENSIDA) [41], it is noteworthy that from all individuals under ART in Mexico, 89% have at least one registered viral load assay after ART initiation, and only 63% individuals with 6 or more months under ART have viral loads under 400 copies/ml [39], [41]. Stratifying these data by state, a negative correlation was found between the prevalence of PI TDR and the proportion of persons with at least one viral load assay registered during ART follow-up (p = 0.0188, r^2^ = 0.4757, data not shown). This suggests an important role of suboptimal clinical follow-up of individuals under ART in HIV TDR spread among the population. On the other hand, several polymorphic ARV drug resistance-associated mutations showed a high frequency in the Mexican cohort compared to other ART-naïve cohorts [7], [38], including T215 revertants and K103R in RT; and A71TV, M46IL and L90M in protease. This could suggest the existence of important founder effects defining polymorphism spread in circulating HIV in Mexico in which other selective pressures such as HLA-mediated immune responses may be involved. Moreover, the fact that some ARV drug resistance-associated mutations were differentially expressed in different geographic areas suggests that several founder effects could be involved in TDR mutation spread in the country. Interestingly, a phylogenetic analysis of the 1655 Mexican viral sequences showed a marked clustering of sequences containing some minor TDR mutations. In particular, distinctive clusters of sequences containing the protease A71V mutation and the RT G333E and K103R mutations were observed, when considering all the sequences together (Figure 4, Figure S2). Several possible transmission pairs were identified in these clusters. Although a geographical effect could be detected within some of the clusters, the inclusion of sequences from distant geographical regions and varied genetic distances in others was also apparent, suggesting a wide circulation of these mutations in Mexico. These data suggest a possible role of viral evolution in TDR spread in Mexico. However, the extent to which different ART management policies, patient follow-up and physician and patient education may influence TDR spread remains to be further assessed.

The current study represents the largest and most comprehensive study to date assessing HIV molecular epidemiology and TDR prevalence in Mexico. We show that TDR prevalence in Mexico remains at an intermediate level. Our data strongly suggests the presence of selection and transmission of TDR mutations in unique and complex patterns within the country. Further and continuous TDR surveillance is necessary to gain more in-depth knowledge on TDR spread and patterns in Mexico, and to confirm the trends observed in this study. Whether regional variations in TDR patterns and trends are associated with differences in ARV drug usage/ART efficacy or with founder events in viral evolution in different geographic areas within the country remains to be further addressed.

## Supporting Information

Figure S1 **Regional transmitted drug resistance (TDR) trends in Mexico.** Approximate dates of infection were estimated using a model described by Mellors et al [33]. TDR fluctuations were examined over the estimated dates of infection by graphical methods using moving average with a six-month window for each geographic region. Significance was assessed with Poisson regression. Center – Mexico City, Morelos, Tlaxcala, Puebla, State of Mexico; East – Veracruz, Quintana Roo; NE – Nuevo León, Guanajuato, Queretaro; NW – Sinaloa, Sonora, Baja California; South – Oaxaca, Guerrero, Chiapas; West – Jalisco, Michoacan.
(TIFF)

Figure S2 **Phylogenetic Maximum Likelihood (ML) tree of the protease (A) and reverse transcriptase (B) nucleotide sequences of 1655 Mexican individuals.** The ML tree was estimated using PhyML ver 2.4.4, with 100 replicates for bootstrap analysis, and a GTR+I+gamma base optimization model. Bootstrap support from 100 replicates is shown for values >50%.
(TIFF)

Table S1 **Ordinal logistic regression model of the effect of CD4+ T cell count and plasma viral load on the Stanford scores of all patients^a^.**
(DOC)

Table S2 **TDR prevalence and demographic/clinical characteristics of a cohort of the Mexican cohort by state.**
(DOC)

Table S3 **ARV drug resistance mutations expressed differentially in viruses from different geographic regions in Mexico.**
(DOC)

## References

[1] (2007). Evidence of a decline in transmitted HIV-1 drug resistance in the United Kingdom.. Aids.

[2] Booth CL, Geretti AM (2007). Prevalence and determinants of transmitted antiretroviral drug resistance in HIV-1 infection.. J Antimicrob Chemother.

[3] Callegaro A, Svicher V, Alteri C, Lo Presti A, Valenti D (2011). Epidemiological network analysis in HIV-1 B infected patients diagnosed in Italy between 2000 and 2008.. Infect Genet Evol.

[4] Cardoso LP, Queiroz BB, Stefani MM (2009). HIV-1 pol phylogenetic diversity and antiretroviral resistance mutations in treatment naive patients from Central West Brazil.. J Clin Virol.

[5] Geretti AM (2007). Epidemiology of antiretroviral drug resistance in drug-naive persons.. Curr Opin Infect Dis.

[6] Hattori J, Shiino T, Gatanaga H, Yoshida S, Watanabe D (2010). Trends in transmitted drug-resistant HIV-1 and demographic characteristics of newly diagnosed patients: nationwide surveillance from 2003 to 2008 in Japan.. Antiviral Res.

[7] Wheeler WH, Ziebell RA, Zabina H, Pieniazek D, Prejean J (2010). Prevalence of transmitted drug resistance associated mutations and HIV-1 subtypes in new HIV-1 diagnoses, U.S.-2006.. Aids.

[8] Jayaraman GC, Archibald CP, Kim J, Rekart ML, Singh AE (2006). A population-based approach to determine the prevalence of transmitted drug-resistant HIV among recent versus established HIV infections: results from the Canadian HIV strain and drug resistance surveillance program.. J Acquir Immune Defic Syndr.

[9] Wong KH, Chan WK, Yam WC, Chen JH, Alvarez-Bognar FR (2010). Stable and low prevalence of transmitted HIV type 1 drug resistance despite two decades of antiretroviral therapy in Hong Kong.. AIDS Res Hum Retroviruses.

[10] Wittkop L, Gunthard HF, de Wolf F, Dunn D, Cozzi-Lepri A (2011). Effect of transmitted drug resistance on virological and immunological response to initial combination antiretroviral therapy for HIV (EuroCoord-CHAIN joint project): a European multicohort study.. Lancet Infect Dis.

[11] Bennett DE, Bertagnolio S, Sutherland D, Gilks CF (2008). The World Health Organization's global strategy for prevention and assessment of HIV drug resistance.. Antivir Ther.

[12] Audelin AM, Lohse N, Obel N, Gerstoft J, Jorgensen LB (2009). The incidence rate of HIV type-1 drug resistance in patients on antiretroviral therapy: a nationwide population-based Danish cohort study 1999–2005.. Antivir Ther.

[13] Payne BA, Nsutebu EF, Hunter ER, Olarinde O, Collini P (2008). Low prevalence of transmitted antiretroviral drug resistance in a large UK HIV-1 cohort.. J Antimicrob Chemother.

[14] Vercauteren J, Wensing AM, van de Vijver DA, Albert J, Balotta C (2009). Transmission of drug-resistant HIV-1 is stabilizing in Europe.. J Infect Dis.

[15] Green H, Tilston P, Fearnhill E, Pillay D, Dunn DT (2008). The impact of different definitions on the estimated rate of transmitted HIV drug resistance in the United Kingdom.. J Acquir Immune Defic Syndr.

[16] Shafer RW, Rhee SY, Bennett DE (2008). Consensus drug resistance mutations for epidemiological surveillance: basic principles and potential controversies.. Antivir Ther.

[17] Shafer RW, Schapiro JM (2008). HIV-1 drug resistance mutations: an updated framework for the second decade of HAART.. AIDS Rev.

[18] CENSIDA (2009). El VIH/SIDA en México 2009.. http://www.censida.salud.gob.mx/descargas/2009/VIHSIDAenMexico2009.pdf.

[19] Bautista-Arredondo S, Dmytraczenko T, Kombe G, Bertozzi SM (2008). Costing of scaling up HIV/AIDS treatment in Mexico.. Salud Publica Mex.

[20] Geretti AM (2006). HIV-1 subtypes: epidemiology and significance for HIV management.. Curr Opin Infect Dis.

[21] Hemelaar J, Gouws E, Ghys PD, Osmanov S (2006). Global and regional distribution of HIV-1 genetic subtypes and recombinants in 2004.. Aids.

[22] LANL (2010). Los Alamos HIV Database..

[23] UNAIDS (2010). UNAIDS Report on the Global AIDS Epidemic 2010.. http://www.unaids.org/globalreport/Global_report.htm.

[24] Vazquez-Valls E, Escoto-Delgadillo M, Lopez-Marquez FC, Castillero-Manzano M, Echegaray-Guerrero E (2010). Molecular epidemiology of HIV type 1 in Mexico: emergence of BG and BF intersubtype recombinants.. AIDS Res Hum Retroviruses.

[25] Eyzaguirre L, Brouwer KC, Nadai Y, Patterson TL, Ramos R (2007). First molecular surveillance report of HIV type 1 in injecting drug users and female sex workers along the U.S.-Mexico border.. AIDS Res Hum Retroviruses.

[26] Rivera-Morales LG, Novitsky VA, Trujillo JR, Lavalle-Montalvo C, Cano-Dominguez C (2001). The molecular epidemiology of HIV type 1 of men in Mexico.. AIDS Res Hum Retroviruses.

[27] Pyne MT, Holzmayer V, Hackett J, Hillyard DR.

[28] CENSIDA (2010). Panorama Epidemiológico del VIH/SIDA en México.. http://www.censida.salud.gob.mx/interior/panorama.html.

[29] Liu TF, Shafer RW (2006). Web resources for HIV type 1 genotypic-resistance test interpretation.. Clin Infect Dis.

[30] Stanford-University (2006). Stanford HIV Drug Resistance Database.. http://sierra2.stanford.edu/sierra/servlet/JSierra.

[31] Bennett DE, Camacho RJ, Otelea D, Kuritzkes DR, Fleury H (2009). Drug resistance mutations for surveillance of transmitted HIV-1 drug-resistance: 2009 update.. PLoS One.

[32] Bertagnolio S, Derdelinckx I, Parker M, Fitzgibbon J, Fleury H (2008). World Health Organization/HIVResNet Drug Resistance Laboratory Strategy.. Antivir Ther.

[33] Mellors JW, Munoz A, Giorgi JV, Margolick JB, Tassoni CJ (1997). Plasma viral load and CD4+ lymphocytes as prognostic markers of HIV-1 infection.. Ann Intern Med.

[34] de Oliveira T, Deforche K, Cassol S, Salminen M, Paraskevis D (2005). An automated genotyping system for analysis of HIV-1 and other microbial sequences.. Bioinformatics.

[35] Bioafrica (2006). http://www.bioafrica.net/subtypetool/html/subtypinghiv.html.

[36] LANL (2011). Los Alamos HIV Database.. http://www.hiv.lanl.gov/content/sequence/RIP/RIP.html.

[37] Paradis E, Claude J, Strimmer K (2004). APE: Analyses of Phylogenetics and Evolution in R language.. Bioinformatics.

[38] Stanford-University (2006). HIV Drug Resistance Database.. http://hivdb.stanford.edu/.

[39] Calva-Mercado J, Vargas-Infante Y, Córdova-Villalobos J, Ponce de León-Rosales S, Valdespino J (2009). Cobertura universal con la terapia antirretroviral combinada. Logros y desafíos en la Secretaría de Salud de México.. 25 años de SIDA en México Logros, Desaciertos y Retos. 2nd Edition.

[40] Magis-Rodriguez C, Lemp G, Hernandez MT, Sanchez MA, Estrada F (2009). Going North: Mexican migrants and their vulnerability to HIV.. J Acquir Immune Defic Syndr.

[41] CENSIDA (2010). Boletín SALVAR.. http://www.censida.salud.gob.mx/interior/atencion/bol_salvar.html.

