# Correction: National Prevalence and Trends of HIV Transmitted Drug Resistance in Mexico

**DOI:** 10.1371/annotation/3797af3f-0573-41bf-8d50-05309fd3d187

**Published:** 2012-07-10

**Authors:** Santiago Avila-Ríos, Claudia García-Morales, Daniela Garrido-Rodríguez, Christopher E. Ormsby, Ramón Hernández-Juan, Jaime Andrade-Villanueva, Luz A. González-Hernández, Indiana Torres-Escobar, Samuel Navarro-Álvarez, Gustavo Reyes-Terán, For the Mexican HIV Molecular Epidemiology Project Group

There is an error in Figure 2. The correct Figure 2 can be seen here: 

**Figure:**
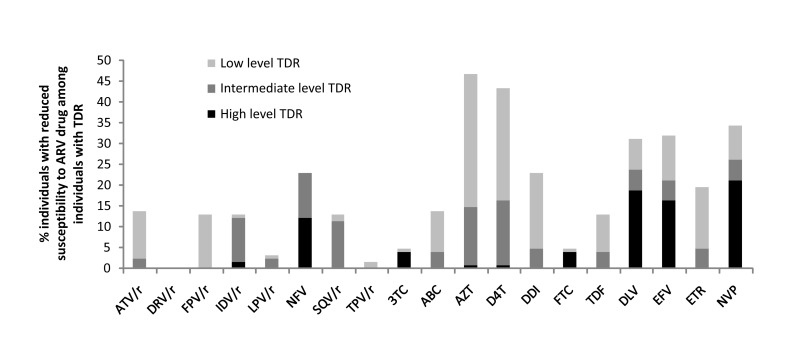


[^] 

