# Supplementary figures and images for: National Prevalence and Trends of HIV Transmitted Drug Resistance in Mexico
Source: PLoS One. 2011 Nov 15;6(11):e27812. doi: 10.1371/journal.pone.0027812 (PMC3217006; doi:10.1371/journal.pone.0027812)

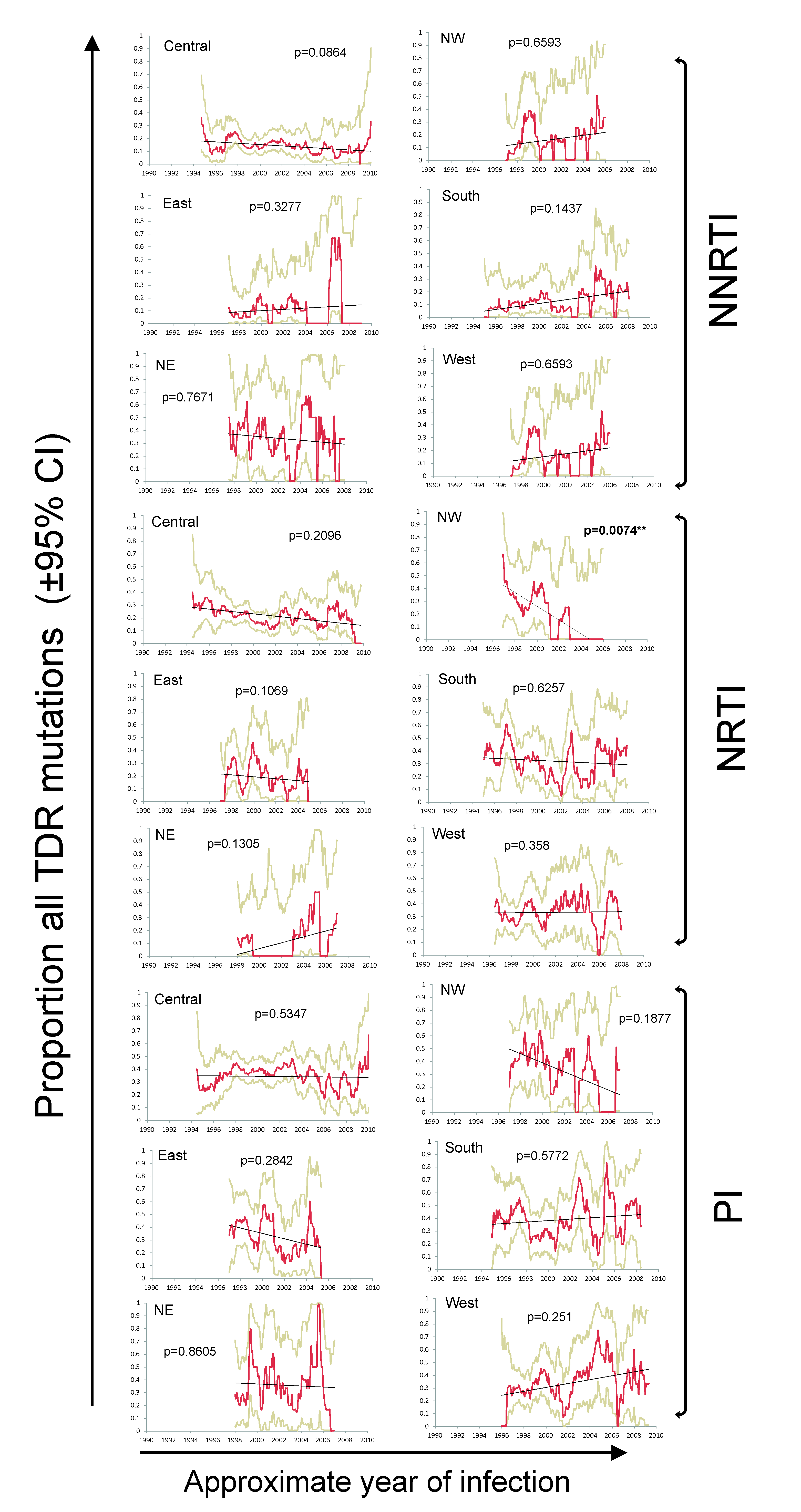

Supplement: Figure S1 — Regional transmitted drug resistance (TDR) trends in Mexico. Approximate dates of infection were estimated using a model described by Mellors et al [33]. TDR fluctuations were examined over the estimated dates of infection by graphical methods using moving average with a six-month window for each geographic region. Significance was assessed with Poisson regression. Center – Mexico City, Morelos, Tlaxcala, Puebla, State of Mexico; East – Veracruz, Quintana Roo; NE – Nuevo León, Guanajuato, Queretaro; NW – Sinaloa, Sonora, Baja California; South – Oaxaca, Guerrero, Chiapas; West – Jalisco, Michoacan. (TIFF) [file pone.0027812.s001.tiff]
