# Supplementary material for: National Prevalence and Trends of HIV Transmitted Drug Resistance in Mexico
Source: PLoS One. 2011 Nov 15;6(11):e27812. doi: 10.1371/journal.pone.0027812 (PMC3217006; doi:10.1371/journal.pone.0027812)
Supplement: Table S1 — Ordinal logistic regression model of the effect of CD4+ T cell count and plasma viral load on the Stanford scores of all patientsa. (DOC) [file pone.0027812.s003.doc]

**Table S1.** Ordinal logistic regression model of the effect of CD4+ T cell count and plasma viral load on the Stanford scores of all patients^a^.

| **Variable** | **Coefficient** | **Standard error** | **Wald Z** | **Odds ratio** | **95% confidence low** | **95% confidence high** | **p value** |
| --- | --- | --- | --- | --- | --- | --- | --- |
| CD4+ count | 5.6e-05 | 0.0002 | 0.25 | 1.02 | 0.88 | 1.18 | 0.8006 |
| Viral load | 0.1434 | 0.0689 | 2.08 | 1.16 | 1.01 | 1.34 | 0.0374 |

^a^Stanford scores were the maximum score reached in any drug. Package Design ver 2.3-0 was used.
