# Supplementary material for: National Prevalence and Trends of HIV Transmitted Drug Resistance in Mexico
Source: PLoS One. 2011 Nov 15;6(11):e27812. doi: 10.1371/journal.pone.0027812 (PMC3217006; doi:10.1371/journal.pone.0027812)
Supplement: Table S2 — TDR prevalence and demographic/clinical characteristics of a cohort of the Mexican cohort by state. (DOC) [file pone.0027812.s004.doc]

**Table S2.** TDR prevalence and demographic/clinical characteristics of a cohort of the Mexican cohort by state.

|  | **State** | | | | | | | | | | | | | |
| --- | --- | --- | --- | --- | --- | --- | --- | --- | --- | --- | --- | --- | --- | --- |
|  | **All** | Baja California | Chiapas | Mexico City | State of Mexico | Jalisco | Guerrero | Morelos | Nuevo Leon | Oaxaca | Puebla | Veracruz | Others | p-value |
| **N** | **1655** | 62 | 67 | 251 | 361 | 255 | 51 | 162 | 61 | 117 | 112 | 123 | 33 |  |
| **Mean Age**  **(years)** | **32.5** | 34.0 | 36.0 | 32.7 | 31.3 | 34.2 | 34.9 | 31.4 | 34.2 | 29.7 | 31.1 | 32.4 | 36.6 | 0.0000 |
| **Proportion of females**  **(%)** | **20.4** | 30.7 | 29.9 | 9.2 | 20.2 | 16.5 | 27.5 | 21.0 | 23.0 | 35.0 | 26.1 | 17.1 | 21.2 | 0.0001 |
| **Median Plasma viral load**  **(log RNA copies/ml)** | **4.84** | 4.90 | 5.25 | 4.91 | 4.86 | 5.10 | 4.48 | 4.75 | 4.58 | 4.69 | 5.06 | 4.76 | 4.38 | 0.0001 |
| **Median CD4+ T cell count (cells/ul)** | **227.8** | 202.5 | 117.4 | 260.8 | 224.0 | 146.3 | 309.3 | 252.1 | 325.2 | 266.0 | 165.0 | 251.0 | 249.9 | 0.0001 |
| **TDR any drug (%)**  **(SS>15)** | **7.4** | 8.1 | 7.5 | 6.0 | 9.1 | 7.1 | 11.8 | 6.2 | 8.2 | 5.1 | 5.4 | 9.8 | 6.1 | 0.888 |
| **PI TDR (%)**  **(SS>15)** | **1.7** | 1.6 | 0.0 | 2.8 | 2.2 | 1.2 | 2.0 | 1.2 | 1.6 | 0.9 | 1.8 | 1.6 | 3.0 | 0.6243 |
| **NRTI TDR (%)**  **(SS>15)** | **4.2** | 6.5 | 3.0 | 2.0 | 5.8 | 4.7 | 5.9 | 3.1 | 8.2 | 2.6 | 2.7 | 4.1 | 3.0 | 0.4051 |
| **NNRTI TDR (%)**  **(SS>15)** | **2.5** | 3.2 | 4.5 | 2.0 | 2.2 | 3.5 | 3.9 | 3.1 | 0.0 | 1.7 | 0.9 | 4.9 | 0.0 | 0.5608 |
| **WHO TDR**  **(%)** | **7.2** | 8.1 | 6.0 | 5.2 | 8.31 | 6.3 | 11.8 | 4.9 | 8.2 | 5.1 | 5.4 | 8.9 | 6.1 | 0.7514 |

SS – Stanford Score
