# Supplementary material for: National Prevalence and Trends of HIV Transmitted Drug Resistance in Mexico
Source: PLoS One. 2011 Nov 15;6(11):e27812. doi: 10.1371/journal.pone.0027812 (PMC3217006; doi:10.1371/journal.pone.0027812)
Supplement: Table S3 — ARV drug resistance mutations expressed differentially in viruses from different geographic regions in Mexico. (DOC) [file pone.0027812.s005.doc]

**Table S3**. ARV drug resistance mutations expressed differentially in viruses from different geographic regions in Mexico.

| **Mutation** | **Geographic**  **Region*** | **Mutation (+) Region (+)** | | **Mutation (–) Region (+)** | | **Mutation (+) Region (-)** | | **Mutation (-) Region (-)** | | **p-value** | **q-value** |
| --- | --- | --- | --- | --- | --- | --- | --- | --- | --- | --- | --- |
|  |  | **n** | **(%)** | **n** | **(%)** | **n** | **(%)** | **n** | **(%)** |  |  |
| K103R | North East | 17/68 | (25.0) | 51/68 | (75.0) | 102/1589 | (6.4) | 1487/1589 | (93.6) | 2.34E-06 | 0.000858081 |
| G333E | West | 35/261 | (13.4) | 226/261 | (86.6) | 93/1396 | (6.7) | 1303/1396 | (93.3) | 0.000543339 | 0.099431002 |
| V179D | Center | 33/889 | (3.7) | 856/889 | (96.3) | 11/768 | (1.4) | 757/768 | (98.6) | 0.00523124 | 0.638211232 |
| V179D | South | 1/237 | (0.4) | 236/237 | (99.6) | 43/1420 | (3.0) | 1377/1420 | (97.0) | 0.015044281 | 0.905132376 |
| K103R | Center | 51/889 | (5.7) | 838/889 | (94.3) | 68/768 | (8.9) | 700/768 | (91.1) | 0.016823015 | 0.905132376 |
| T69S | North West | 5/84 | (6.0) | 79/84 | (94.0) | 26/1573 | (1.7) | 1547/1573 | (98.3) | 0.017796493 | 0.905132376 |
| G333D | South | 2/237 | (0.8) | 235/237 | (99.2) | 0/1420 | (0.0) | 1420/1420 | (100.0) | 0.020383441 | 0.905132376 |
| M184VI | Center | 0/889 | (0.0) | 889/889 | (100.0) | 5/768 | (0.7) | 763/768 | (99.3) | 0.021239826 | 0.905132376 |
| K219QEN | North East | 2/68 | (2.9) | 66/68 | (97.1) | 4/1589 | (0.3) | 1585/1589 | (99.7) | 0.022371475 | 0.905132376 |
| K238T | West | 2/261 | (0.8) | 259/261 | (99.2) | 0/1396 | (0.0) | 1396/1396 | (100.0) | 0.024730393 | 0.905132376 |
| A71IVT | South | 72/237 | (30.4) | 165/237 | (69.6) | 335/1420 | (23.6) | 1085/1420 | (76.4) | 0.027700283 | 0.921663961 |
| K219QEN | North West | 2/84 | (2.4) | 82/84 | (97.6) | 4/1573 | (0.3) | 1569/1573 | (99.7) | 0.033347106 | 1 |
| K70R | North East | 1/68 | (1.5) | 67/68 | (98.5) | 0/1589 | (0.0) | 1589/1589 | (100.0) | 0.041038021 | 1 |
| L33F | North West | 2/84 | (2.4) | 82/84 | (97.6) | 5/1573 | (0.3) | 1568/1573 | (99.7) | 0.045167783 | 1 |

*Center – Mexico City, Morelos, Tlaxcala, Puebla, State of Mexico; East – Veracruz, Quintana Roo; North East – Nuevo León, Guanajuato, Queretaro; North West – Sinaloa, Sonora, Baja California; South – Oaxaca, Guerrero, Chiapas; West – Jalisco, Michoacan.
